# Synthesis and structure of di­chlorido­{(*E*)-4-methyl-*N*′-[1-(pyridin-2-yl)ethyl­idene]benzohydrazide}zinc(II)

**DOI:** 10.1107/S2056989026008674

**Published:** 2026-09-08

**Authors:** Mohamedou El Boukhary, Thierno Moussa Seck, Farba Bouyagui Tamboura, Ibrahima Elhadji Thiam, Aliou Hamady Barry, Pascal Retailleau, Mohamed Gaye

**Affiliations:** a Equipe de recherche en Chimie des matériaux Inorganiques et Organiques (ECMIO), Département de Chimie, Unité de Formation et de Recherche, Université Alioune Diop, Bambey, Senegal; b Unité de chimie des matériaux (ChimMat), Département de Chimie, Faculté des Sciences et Techniques, Université de Nouakchott, Nouakchott, Mauritania; c Département de Chimie Faculté des Sciences et Techniques Université Cheik Anta Diop Dakar Senegal; d Substances Naturelles, CNRS UPR 2301, Université Paris-Sud, Université Paris-Saclay, 1 av. de la Terrasse, 91198 Gif-sur-Yvette, France; University of Aberdeen, United Kingdom

**Keywords:** methyl­benzohydrazone, 2-acetyl­pyridine, crystal structure

## Abstract

The ligand in the title complex, [ZnCl_2_(C_15_H_15_N_3_O)], was obtained by a condensation reaction between *p*-tolyl hydrazide and 2-acetyl­pyridine. The ligand mol­ecule bonds to the metal ion in a tridentate manner *via* its imino nitro­gen atom, its carbonyl oxygen atom, and the nitro­gen atom of the pyridine ring. The geometry of the coordination polyhedron around the zinc ion is inter­mediate between a trigonal bipyramid and a square pyramid, but closer to the latter. In the extended structure, pairwise N—H⋯Cl hydrogen bonds generate inversion dimers and very weak C—H⋯Cl inter­actions link the dimers into sheets.

## Abstract 2

The ligand in the title complex was obtained by a condensation reaction between *p*-tolyl hydrazide and 2-acetyl­pyridine. The ligand mol­ecule bonds to the metal ion in a tridentate manner *via* its imino nitro­gen atom, its carbonyl oxygen atom, and the nitro­gen atom of the pyridine ring. In the extended structure, pairwise N—H⋯Cl hydrogen bonds generate inversion dimers and very weak C—H⋯Cl inter­actions link the dimers into sheets.

## 1. Chemical context

In recent years, hydrazone derivatives containing a –C(=O)NHN=CH– moiety have attracted the attention of many chemists due to their diverse biological properties and large range of applications in medicinal chemistry (Mansi *et al.*, 2023 ▸; Socea *et al.*, 2022 ▸; Mali *et al.*, 2021 ▸). In their formal structures, hydrazine derivatives exhibit amido–iminol tautomerism (Polo-Cerón *et al.*, 2021 ▸; Jamadar *et al.*, 2012 ▸), with the amide form predominating in the solid state (Gamov *et al.*, 2019 ▸). Theoretically, acyl­hydrazone derivatives have four isomers, two of which are geometric isomers (*E*/*Z*) due to the rigid C=N double bond, and two are conformational isomers (*syn*/*anti*) due to rotation about the single N—N bond (Chen *et al.*, 2019 ▸; Dasgupta *et al.*, 2020 ▸). In a mixture of *E* and *Z* isomers, it has been shown that the *E* isomer predominates (Naskar *et al.*, 2011 ▸). In coordination chemistry, hydrazone-derived Schiff bases are effective ligands due to their ability to chelate metal ions and to lead to the formation of supra­molecular structures *via* inter­molecular inter­actions (Mondal *et al.*, 2013 ▸). Depending on reaction conditions such as pH, ligand concentration and the oxidation state of the metal ion involved, these types of Schiff bases coordinate with metal ions in their neutral amide form or in their imino­late form. As part of our studies in this area, we now describe the synthesis and structure of the title compound, [Zn(C_15_H_15_N_3_O)Cl_2_] (**I**).
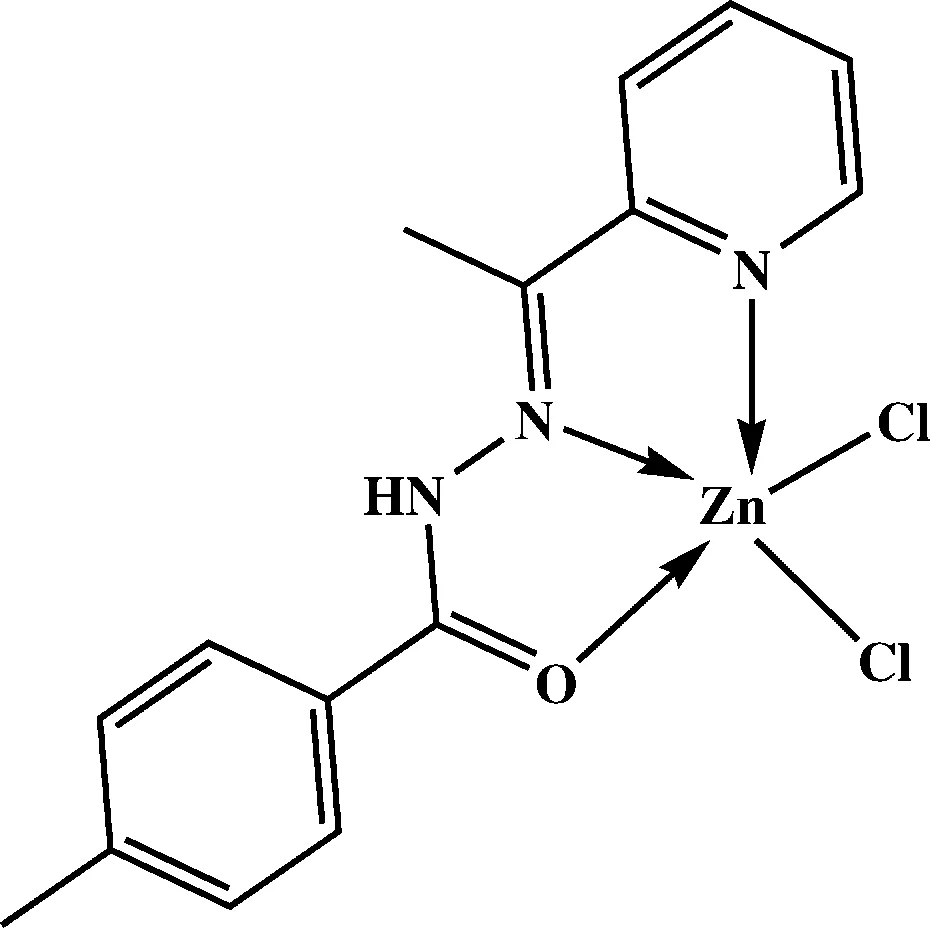


## 2. Structural commentary

**Figure 1:**
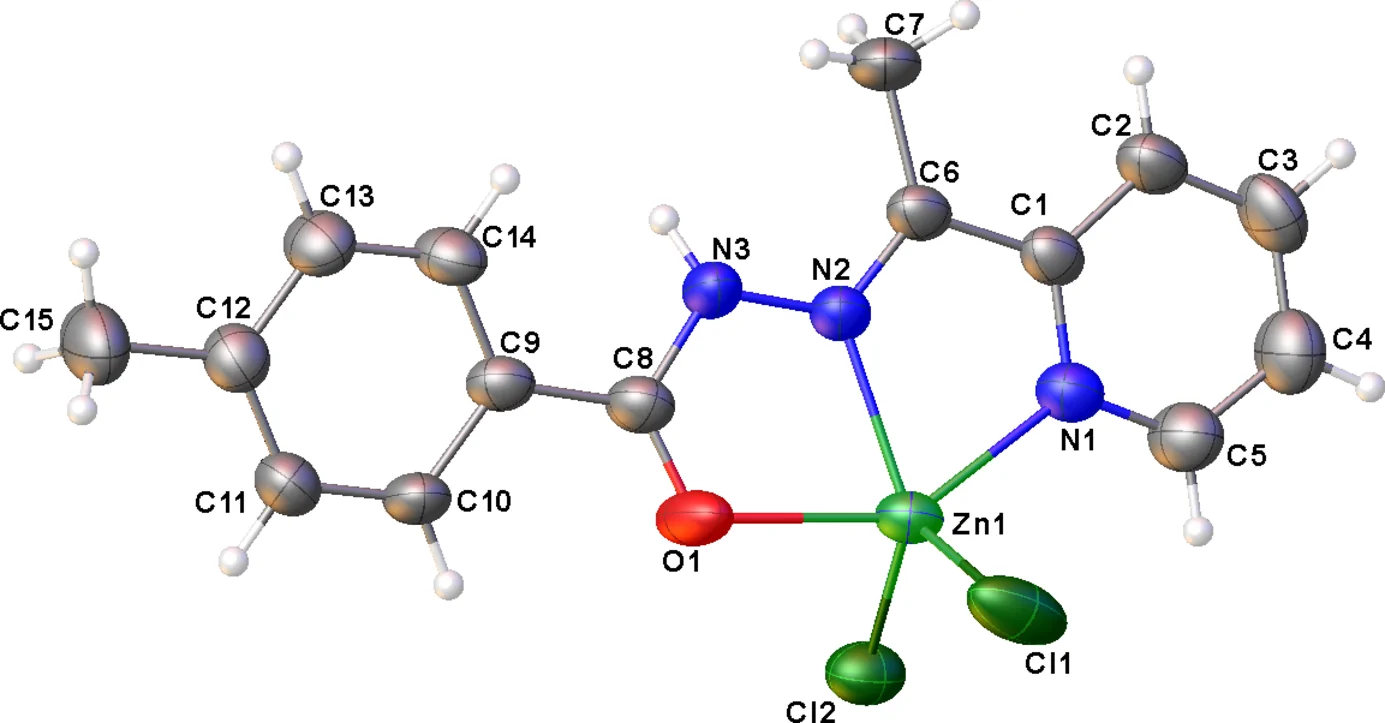
The mol­ecular structure of (**I**) with displacement ellipsoids plotted at the 30% probability level.

Compound (**I**) crystallizes in the triclinic system with space group *P*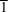. The mol­ecular structure of (**I**) (Fig. 1 ▸) reveals a mononuclear complex in which the Zn^2+^ metal ion is penta­coordinated by a tridentate N,N,O-bonded ligand and two chloride ions: selected geometrical data are presented in Table 1 ▸. The bond angles around the metal ion reveal a notable distortion in the coordination sphere, particularly the bite angles N2—Zn1—N1 and N2—Zn1—O1, which are characteristic of the constraint imposed by chelation. The N1/C1/C6/N2/Zn1 chelate ring is a shallow envelope with Zn1 as the flap deviating by −0.228 (1) Å from the other atoms whereas the N2/N3/C8/O1/Zn1 ring is almost planar (r.m.s. deviation = 0.018 Å). The Addison τ parameter (Addison *et al.*, 1984 ▸) of 0.29 for the metal ion suggests a geometry inter­mediate between trigonal-pyramidal and square-based pyramidal but closer to the latter. Analysis of the ligand bond lengths confirms its coordination to the metal in the amide form. The C8=O1 distance of 1.233 (6) Å indicates that the C=O bond retains its double-bond character, ruling out imino­lization. The distances N2—N3 = 1.370 (5) Å and C6—N2 = 1.287 (5) Å reflect electronic delocalization within the hydrazone fragment, while the nearly planar nature of the chelating system promotes π conjugation. Overall, the complex mol­ecule has approximate local *C*_s_ symmetry.

## 3. Supra­molecular features

**Figure 2:**
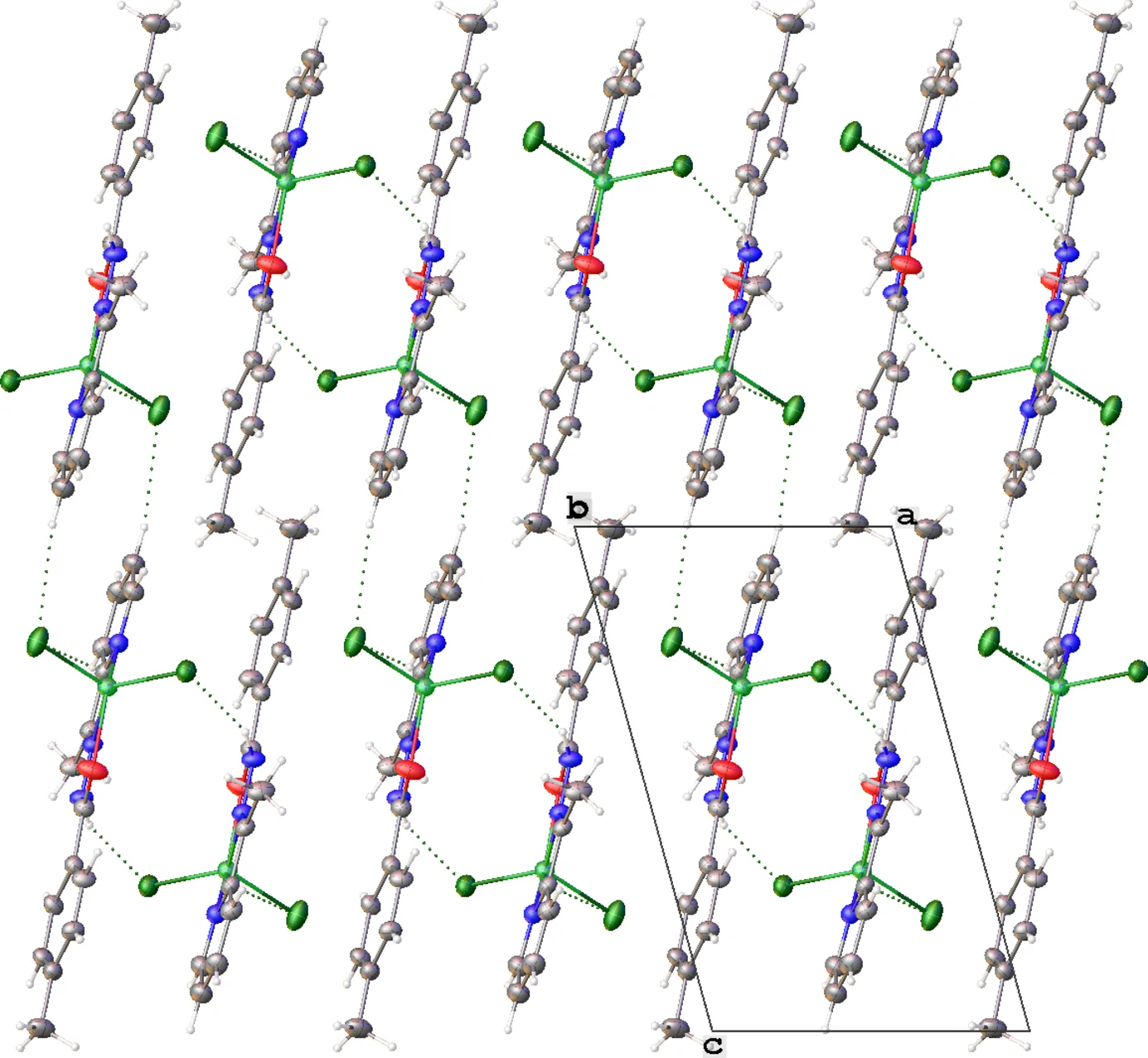
Hydrogen-bonded sheets lying parallel to the *bc* plane.

In the extended structure of (**I**), pairwise N3—H3⋯Cl2 hydrogen bonds (Table 2 ▸) generate inversion dimers with an 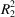
(10) graph-set descriptor. The dimers are linked by very weak C—H⋯Cl hydrogen bonds to form sheets lying parallel to the *bc* plane, which are further linked into a three-dimensional network (Figs. 2 ▸ and 3 ▸).

## 4. Database survey

We are not aware of any previous reports of the ligand reported here but similar ligands possessing O,N,N donor groups exist in the literature. For general background to hydrazones and their complexes, see Abubakar & Habu (2026 ▸). For similar ligands and their Ni^II^, Zn^II^, and V^V^ complexes, see Laila *et al.* (2026 ▸) and You *et al.* (2025 ▸).

## 5. Synthesis and crystallization

*p*-Tolyl hydrazide (1.5 g, 10 mmol) and 2-acetyl­pyridine in a 1:1 ratio in methanol, in the presence of a few drops of glacial acetic acid were heated to reflux for seven hours. After cooling and filtration, a white solid was obtained in 79% yield (m.p. = 449–451 K). FT-IR (ν, cm^−1^): 3236 (NH); 1668 (C=O); 1609 (C=N) imine; 1525–1434 (C=C) aromatic; 1580 (C=N) pyridine; 1122 (N—N). ^1^H NMR [CDCl_3_, δ (ppm)]: 2.52 [3H, *s*, CH_3_—C=N)pyridine]); 2.56 (3H, *s*, CH_3_—Ar); 7.42 (2H, *d*, H—Ar); 7.66 (2H, *d*, H—Ar); 7.75 (1H, *d*, H—Ar); 7.91 (1H, *d*, H—Ar); 8.62 (1H, *m*, H—Ar); 8.75 (1H, *dd*, H—pyridine); 15.73 (1H, *s*, H—N). ^13^C NMR [CDCl_3_, δ (ppm)]: 164.47 (C=O); 153.55 (C=N), 147.38 (C—Ar); 142.92 (C—Ar); 142.28 (C—Ar); 137.97 (C—Ar); 131.14 (C—Ar); 129.36 (C—Ar); 127.64 (C—Ar); 124.20 (C—Ar); 124.06 (C—Ar); 22.65 (CH_3_—Ar); 21.55 (CH_3_—Ar). Calculated analysis for C_15_H_15_N_3_O: C, 71.13; H, 5.97; N, 16.59%; found C, 71.18; H, 6.04; N, 16.63%. To a solution of the ligand (0.10 g, 0.4 mmol) in 15 ml of methanol, 0.4 mmol of ZnCl_2_ was added. The mixture was refluxed for two h. Upon cooling, the resulting solution was filtered, and the filtrate was left to evaporate slowly at 298 K. After one week, colorless crystals of (**I**) suitable for X-ray diffraction were collected.

## 6. Refinement

Crystal data, data collection and structure refinement details are summarized in Table 3 ▸. H atoms attached to the amide group were located from difference-Fourier maps and refined. Other H atoms (CH, CH_3_ groups) were geometrically optimized and refined as riding atoms with *U*_iso_(H) = 1.2*U*_eq_(C) (1.5 for CH_3_).

## Supplementary Material

Crystal structure: contains datablock(s) I. DOI: 10.1107/S2056989026008674/hb8248sup1.cif

Structure factors: contains datablock(s) I. DOI: 10.1107/S2056989026008674/hb8248Isup2.hkl

CCDC reference: 2580323

Additional supporting information:  crystallographic information; 3D view; checkCIF report

## Figures and Tables

**Figure 3:**
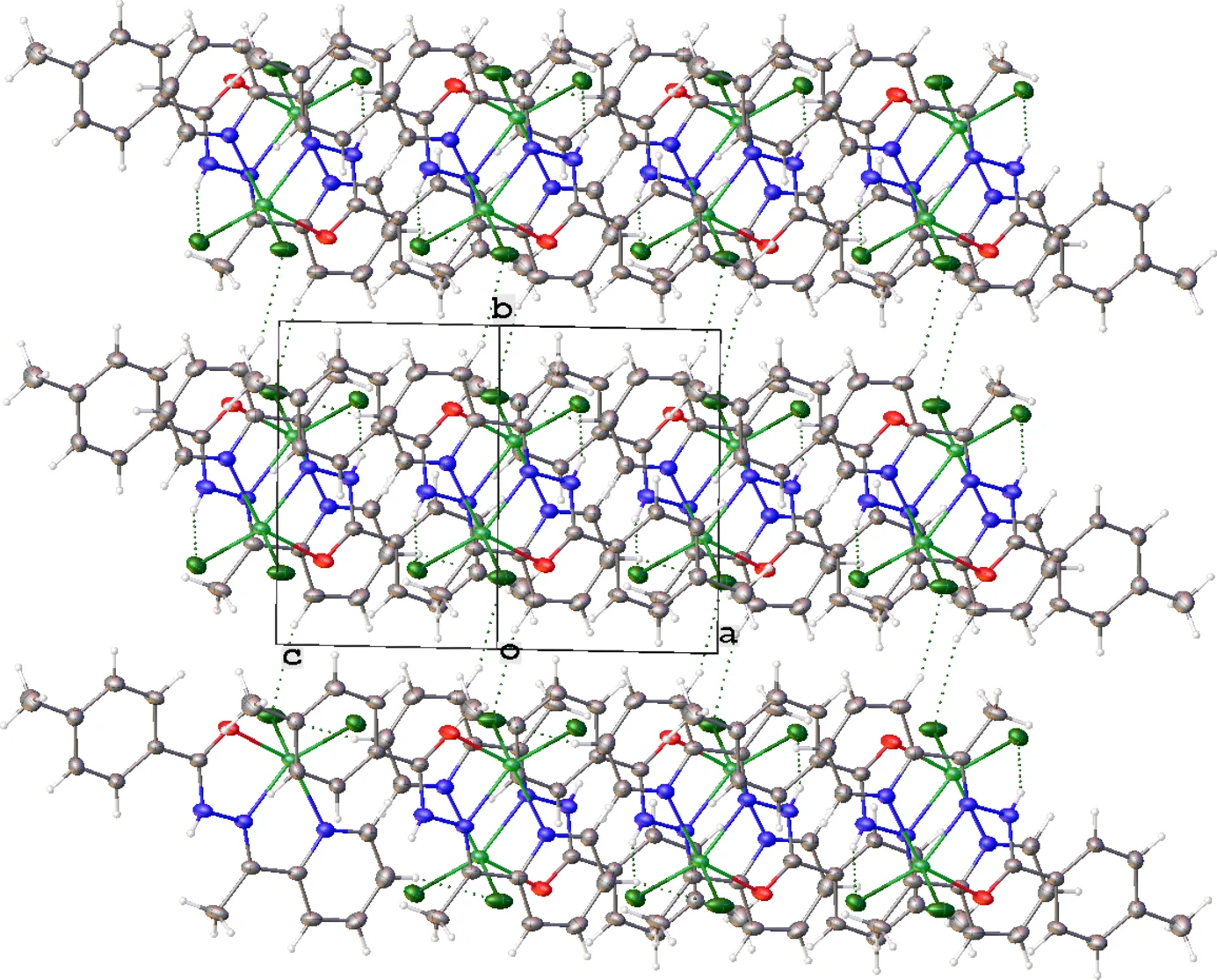
Hydrogen-bonded sheets lying parallel to the *ac* plane.

**Table 1:** Selected geometric parameters (Å, °)

Zn1—Cl2	2.2348 (12)	Zn1—O1	2.260 (3)
Zn1—Cl1	2.2318 (13)	Zn1—N2	2.107 (3)
Zn1—N1	2.186 (4)		
			
Cl2—Zn1—O1	94.84 (11)	N1—Zn1—O1	145.35 (13)
Cl1—Zn1—Cl2	111.46 (5)	N2—Zn1—Cl2	127.98 (11)
Cl1—Zn1—O1	98.16 (13)	N2—Zn1—Cl1	120.03 (11)
N1—Zn1—Cl2	103.12 (11)	N2—Zn1—N1	73.64 (13)
N1—Zn1—Cl1	102.32 (11)	N2—Zn1—O1	71.95 (12)

**Table 2:** Hydrogen-bond geometry (Å, °)

*D*—H⋯*A*	*D*—H	H⋯*A*	*D*⋯*A*	*D*—H⋯*A*
N3—H3⋯Cl2^i^	0.87 (2)	2.40 (3)	3.214 (4)	156 (5)
C2—H2⋯Cl1^ii^	0.93	2.93	3.649 (5)	136
C4—H4⋯Cl1^iii^	0.93	2.98	3.888 (6)	165

Symmetry codes: (i) 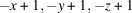; (ii) 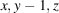; (iii) 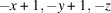.

**Table 3:** Experimental details

Crystal data	
Chemical formula	[ZnCl_2_(C_15_H_15_N_3_O)]
*M*_r_	389.57
Crystal system, space group	Triclinic, *P* 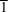
Temperature (K)	293
*a*, *b*, *c* (Å)	7.7318 (6), 8.4484 (8), 12.8117 (11)
α, β, γ (°)	84.016 (7), 74.425 (7), 86.261 (7)
*V* (Å^3^)	801.15 (12)
*Z*	2
Radiation type	Mo *K*α
μ (mm^−1^)	1.87
Crystal size (mm)	0.26 × 0.03 × 0.02

Data collection	
Diffractometer	XtaLAB AFC12 (RINC): Kappa single
Absorption correction	Gaussian (*CrysAlis PRO*; Rigaku OD, 2023 ▸)
*T*_min_, *T*_max_	0.598, 1.000
No. of measured, independent and observed [*I* > 2σ(*I*)] reflections	19173, 3265, 2757
*R*_int_	0.047
(sin θ/λ)_max_ (Å^−1^)	0.625

Refinement	
*R*[*F*^2^ > 2σ(*F*^2^)], *wR*(*F*^2^), *S*	0.056, 0.169, 1.06
No. of reflections	3265
No. of parameters	204
No. of restraints	1
H-atom treatment	H atoms treated by a mixture of independent and constrained refinement
Δρ_max_, Δρ_min_ (e Å^−3^)	1.54, −0.73

Computer programs: *CrysAlis PRO*a (Rigaku OD, 2023 ▸), *SHELXT* (Sheldrick, 2015*a* ▸), *SHELXL2018/3* (Sheldrick, 2015*b* ▸), *OLEX2* 1.3 (Dolomanov *et al.*, 2009 ▸).

## supplementary crystallographic information

### Dichlorido{(E)-4-methyl-N'-[1-(pyridin-2-yl)ethylidene]benzohydrazide}zinc(II) Crystal data

**Table:**

[ZnCl_2_(C_15_H_15_N_3_O)]	*Z* = 2
*M**_r_* = 389.57	*F*(000) = 396.000
Triclinic, *P*1	*D*_x_ = 1.615 Mg m^−^^3^
*a* = 7.7318 (6) Å	Mo *K*α radiation, λ = 0.71073 Å
*b* = 8.4484 (8) Å	Cell parameters from 6354 reflections
*c* = 12.8117 (11) Å	θ = 3.7–29.1°
α = 84.016 (7)°	µ = 1.87 mm^−^^1^
β = 74.425 (7)°	*T* = 293 K
γ = 86.261 (7)°	Prismatic, yellow
*V* = 801.15 (12) Å^3^	0.26 × 0.03 × 0.02 mm

### Dichlorido{(E)-4-methyl-N'-[1-(pyridin-2-yl)ethylidene]benzohydrazide}zinc(II) Data collection

**Table:**

XtaLAB AFC12 (RINC): Kappa single diffractometer	3265 independent reflections
Radiation source: micro-focus sealed X-ray tube, Rigaku (Mo) X-ray Source	2757 reflections with *I* > 2σ(*I*)
Mirror monochromator	*R*_int_ = 0.047
Detector resolution: 5.8140 pixels mm^-1^	θ_max_ = 26.4°, θ_min_ = 3.7°
ω scans	*h* = −9→9
Absorption correction: gaussian (CrysAlisPro; Rigaku OD, 2023)	*k* = −10→10
*T*_min_ = 0.598, *T*_max_ = 1.000	*l* = −15→15
19173 measured reflections	

### Dichlorido{(E)-4-methyl-N'-[1-(pyridin-2-yl)ethylidene]benzohydrazide}zinc(II) Refinement

**Table:**

Refinement on *F*^2^	Primary atom site location: dual
Least-squares matrix: full	Hydrogen site location: mixed
*R*[*F*^2^ > 2σ(*F*^2^)] = 0.056	H atoms treated by a mixture of independent and constrained refinement
*wR*(*F*^2^) = 0.169	*w* = 1/[σ^2^(*F*_o_^2^) + (0.1117*P*)^2^ + 0.6932*P*] where *P* = (*F*_o_^2^ + 2*F*_c_^2^)/3
*S* = 1.06	(Δ/σ)_max_ < 0.001
3265 reflections	Δρ_max_ = 1.54 e Å^−^^3^
204 parameters	Δρ_min_ = −0.73 e Å^−^^3^
1 restraint	

### Dichlorido{(E)-4-methyl-N'-[1-(pyridin-2-yl)ethylidene]benzohydrazide}zinc(II) Special details

**Table:**

Geometry. All esds (except the esd in the dihedral angle between two l.s. planes) are estimated using the full covariance matrix. The cell esds are taken into account individually in the estimation of esds in distances, angles and torsion angles; correlations between esds in cell parameters are only used when they are defined by crystal symmetry. An approximate (isotropic) treatment of cell esds is used for estimating esds involving l.s. planes.

### Dichlorido{(E)-4-methyl-N'-[1-(pyridin-2-yl)ethylidene]benzohydrazide}zinc(II) Fractional atomic coordinates and isotropic or equivalent isotropic displacement parameters (Å^2^)

**Table:**

	*x*	*y*	*z*	*U*_iso_*/*U*_eq_	
Zn1	0.38989 (7)	0.64352 (5)	0.31892 (4)	0.0429 (2)	
Cl2	0.65189 (16)	0.76112 (14)	0.28753 (10)	0.0557 (3)	
Cl1	0.2085 (2)	0.77878 (14)	0.22910 (14)	0.0738 (5)	
N1	0.4632 (5)	0.4296 (4)	0.2319 (3)	0.0448 (8)	
O1	0.2718 (6)	0.7383 (4)	0.4828 (3)	0.0691 (11)	
N2	0.2932 (5)	0.4499 (4)	0.4327 (3)	0.0418 (8)	
N3	0.2126 (6)	0.4816 (4)	0.5379 (3)	0.0483 (9)	
H3	0.214 (7)	0.411 (5)	0.592 (3)	0.058*	
C1	0.3935 (6)	0.2944 (5)	0.2879 (3)	0.0416 (9)	
C9	0.1303 (6)	0.6744 (5)	0.6711 (3)	0.0419 (9)	
C14	0.0322 (6)	0.5671 (5)	0.7500 (4)	0.0479 (10)	
H14	0.018369	0.464954	0.733199	0.058*	
C6	0.2975 (6)	0.3083 (5)	0.4033 (3)	0.0426 (9)	
C8	0.2115 (6)	0.6369 (5)	0.5581 (4)	0.0457 (9)	
C10	0.1503 (7)	0.8272 (5)	0.6982 (4)	0.0500 (10)	
H10	0.216923	0.900526	0.646215	0.060*	
C2	0.4114 (7)	0.1528 (5)	0.2398 (4)	0.0518 (11)	
H2	0.360613	0.060866	0.279332	0.062*	
C11	0.0715 (7)	0.8687 (6)	0.8015 (4)	0.0531 (11)	
H11	0.085523	0.970784	0.818279	0.064*	
C13	−0.0457 (6)	0.6108 (6)	0.8540 (4)	0.0518 (11)	
H13	−0.111027	0.537309	0.906550	0.062*	
C5	0.5538 (7)	0.4239 (6)	0.1288 (4)	0.0565 (11)	
H5	0.603320	0.516982	0.090251	0.068*	
C12	−0.0277 (6)	0.7636 (6)	0.8810 (4)	0.0487 (10)	
C7	0.2165 (9)	0.1664 (6)	0.4756 (4)	0.0639 (14)	
H7A	0.307863	0.105710	0.502040	0.096*	
H7B	0.165939	0.101230	0.435135	0.096*	
H7C	0.123837	0.201349	0.536005	0.096*	
C3	0.5058 (8)	0.1507 (6)	0.1322 (4)	0.0609 (13)	
H3A	0.519876	0.056798	0.098317	0.073*	
C4	0.5788 (8)	0.2870 (7)	0.0753 (4)	0.0639 (13)	
H4	0.643393	0.287701	0.002708	0.077*	
C15	−0.1136 (9)	0.8107 (8)	0.9938 (4)	0.0754 (17)	
H15A	−0.022367	0.818845	1.030978	0.113*	
H15B	−0.196337	0.731666	1.033128	0.113*	
H15C	−0.176888	0.911770	0.989505	0.113*	

### Dichlorido{(E)-4-methyl-N'-[1-(pyridin-2-yl)ethylidene]benzohydrazide}zinc(II) Atomic displacement parameters (Å^2^)

**Table:**

	*U*^11^	*U*^22^	*U*^33^	*U*^12^	*U*^13^	*U*^23^
Zn1	0.0526 (3)	0.0286 (3)	0.0467 (3)	−0.00524 (19)	−0.0127 (2)	0.00132 (19)
Cl2	0.0564 (7)	0.0472 (6)	0.0653 (7)	−0.0115 (5)	−0.0206 (5)	0.0042 (5)
Cl1	0.0821 (10)	0.0370 (6)	0.1211 (13)	−0.0027 (6)	−0.0620 (9)	0.0011 (7)
N1	0.050 (2)	0.0357 (18)	0.0482 (19)	−0.0026 (15)	−0.0133 (16)	−0.0020 (15)
O1	0.108 (3)	0.0326 (17)	0.0500 (18)	−0.0117 (17)	0.0096 (18)	−0.0026 (14)
N2	0.058 (2)	0.0295 (16)	0.0388 (17)	−0.0022 (14)	−0.0146 (15)	−0.0004 (13)
N3	0.076 (3)	0.0322 (18)	0.0353 (17)	−0.0046 (17)	−0.0121 (17)	−0.0009 (14)
C1	0.050 (2)	0.032 (2)	0.047 (2)	0.0004 (16)	−0.0218 (18)	−0.0030 (16)
C9	0.046 (2)	0.031 (2)	0.046 (2)	−0.0016 (16)	−0.0102 (17)	0.0012 (16)
C14	0.048 (2)	0.037 (2)	0.055 (2)	−0.0086 (18)	−0.0046 (19)	−0.0055 (18)
C6	0.056 (2)	0.0298 (19)	0.046 (2)	−0.0024 (17)	−0.0211 (18)	0.0012 (16)
C8	0.056 (3)	0.031 (2)	0.048 (2)	−0.0012 (17)	−0.0109 (19)	−0.0017 (17)
C10	0.074 (3)	0.030 (2)	0.044 (2)	−0.0095 (19)	−0.011 (2)	0.0032 (17)
C2	0.070 (3)	0.032 (2)	0.057 (3)	−0.0008 (19)	−0.023 (2)	−0.0031 (19)
C11	0.070 (3)	0.040 (2)	0.050 (2)	−0.008 (2)	−0.016 (2)	−0.0074 (19)
C13	0.050 (2)	0.051 (3)	0.049 (2)	−0.010 (2)	−0.0015 (19)	−0.004 (2)
C5	0.059 (3)	0.050 (3)	0.055 (3)	−0.002 (2)	−0.005 (2)	−0.005 (2)
C12	0.049 (2)	0.050 (3)	0.048 (2)	−0.0049 (19)	−0.0111 (19)	−0.0080 (19)
C7	0.104 (4)	0.034 (2)	0.053 (3)	−0.018 (2)	−0.020 (3)	0.006 (2)
C3	0.072 (3)	0.048 (3)	0.066 (3)	0.009 (2)	−0.019 (3)	−0.025 (2)
C4	0.064 (3)	0.070 (4)	0.054 (3)	0.004 (3)	−0.008 (2)	−0.016 (2)
C15	0.095 (4)	0.074 (4)	0.054 (3)	−0.017 (3)	−0.004 (3)	−0.020 (3)

### Dichlorido{(E)-4-methyl-N'-[1-(pyridin-2-yl)ethylidene]benzohydrazide}zinc(II) Geometric parameters (Å, º)

**Table:**

Zn1—Cl2	2.2348 (12)	C10—H10	0.9300
Zn1—Cl1	2.2318 (13)	C10—C11	1.371 (7)
Zn1—N1	2.186 (4)	C2—H2	0.9300
Zn1—O1	2.260 (3)	C2—C3	1.378 (7)
Zn1—N2	2.107 (3)	C11—H11	0.9300
N1—C1	1.346 (6)	C11—C12	1.377 (7)
N1—C5	1.323 (6)	C13—H13	0.9300
O1—C8	1.233 (6)	C13—C12	1.396 (7)
N2—N3	1.370 (5)	C5—H5	0.9300
N2—C6	1.287 (5)	C5—C4	1.382 (8)
N3—H3	0.87 (2)	C12—C15	1.503 (7)
N3—C8	1.363 (5)	C7—H7A	0.9600
C1—C6	1.478 (6)	C7—H7B	0.9600
C1—C2	1.385 (6)	C7—H7C	0.9600
C9—C14	1.381 (6)	C3—H3A	0.9300
C9—C8	1.474 (6)	C3—C4	1.365 (8)
C9—C10	1.400 (6)	C4—H4	0.9300
C14—H14	0.9300	C15—H15A	0.9600
C14—C13	1.385 (7)	C15—H15B	0.9600
C6—C7	1.497 (6)	C15—H15C	0.9600
			
Cl2—Zn1—O1	94.84 (11)	C11—C10—C9	120.0 (4)
Cl1—Zn1—Cl2	111.46 (5)	C11—C10—H10	120.0
Cl1—Zn1—O1	98.16 (13)	C1—C2—H2	120.6
N1—Zn1—Cl2	103.12 (11)	C3—C2—C1	118.7 (5)
N1—Zn1—Cl1	102.32 (11)	C3—C2—H2	120.6
N1—Zn1—O1	145.35 (13)	C10—C11—H11	119.1
N2—Zn1—Cl2	127.98 (11)	C10—C11—C12	121.9 (4)
N2—Zn1—Cl1	120.03 (11)	C12—C11—H11	119.1
N2—Zn1—N1	73.64 (13)	C14—C13—H13	119.5
N2—Zn1—O1	71.95 (12)	C14—C13—C12	120.9 (4)
C1—N1—Zn1	115.1 (3)	C12—C13—H13	119.5
C5—N1—Zn1	126.4 (3)	N1—C5—H5	118.3
C5—N1—C1	118.2 (4)	N1—C5—C4	123.4 (5)
C8—O1—Zn1	115.2 (3)	C4—C5—H5	118.3
N3—N2—Zn1	117.7 (2)	C11—C12—C13	118.0 (4)
C6—N2—Zn1	121.3 (3)	C11—C12—C15	121.3 (5)
C6—N2—N3	120.8 (4)	C13—C12—C15	120.7 (5)
N2—N3—H3	121 (4)	C6—C7—H7A	109.5
C8—N3—N2	115.3 (3)	C6—C7—H7B	109.5
C8—N3—H3	117 (4)	C6—C7—H7C	109.5
N1—C1—C6	115.4 (4)	H7A—C7—H7B	109.5
N1—C1—C2	121.7 (4)	H7A—C7—H7C	109.5
C2—C1—C6	122.9 (4)	H7B—C7—H7C	109.5
C14—C9—C8	122.9 (4)	C2—C3—H3A	120.1
C14—C9—C10	118.9 (4)	C4—C3—C2	119.8 (4)
C10—C9—C8	118.2 (4)	C4—C3—H3A	120.1
C9—C14—H14	119.8	C5—C4—H4	121.0
C9—C14—C13	120.3 (4)	C3—C4—C5	118.1 (5)
C13—C14—H14	119.8	C3—C4—H4	121.0
N2—C6—C1	113.7 (4)	C12—C15—H15A	109.5
N2—C6—C7	125.3 (4)	C12—C15—H15B	109.5
C1—C6—C7	121.0 (4)	C12—C15—H15C	109.5
O1—C8—N3	119.8 (4)	H15A—C15—H15B	109.5
O1—C8—C9	123.5 (4)	H15A—C15—H15C	109.5
N3—C8—C9	116.7 (4)	H15B—C15—H15C	109.5
C9—C10—H10	120.0		
			
Zn1—N1—C1—C6	−7.2 (5)	C14—C9—C8—O1	166.9 (5)
Zn1—N1—C1—C2	172.8 (3)	C14—C9—C8—N3	−11.0 (7)
Zn1—N1—C5—C4	−172.6 (4)	C14—C9—C10—C11	−0.6 (7)
Zn1—O1—C8—N3	−2.7 (6)	C14—C13—C12—C11	−0.7 (7)
Zn1—O1—C8—C9	179.5 (3)	C14—C13—C12—C15	179.6 (5)
Zn1—N2—N3—C8	−2.8 (5)	C6—N2—N3—C8	−177.9 (4)
Zn1—N2—C6—C1	6.6 (5)	C6—C1—C2—C3	−178.9 (4)
Zn1—N2—C6—C7	−173.8 (4)	C8—C9—C14—C13	−177.9 (4)
N1—C1—C6—N2	0.8 (5)	C8—C9—C10—C11	177.7 (4)
N1—C1—C6—C7	−178.8 (4)	C10—C9—C14—C13	0.4 (7)
N1—C1—C2—C3	1.1 (7)	C10—C9—C8—O1	−11.4 (7)
N1—C5—C4—C3	0.0 (9)	C10—C9—C8—N3	170.7 (4)
N2—N3—C8—O1	3.7 (7)	C10—C11—C12—C13	0.4 (8)
N2—N3—C8—C9	−178.4 (4)	C10—C11—C12—C15	−179.9 (5)
N3—N2—C6—C1	−178.5 (4)	C2—C1—C6—N2	−179.2 (4)
N3—N2—C6—C7	1.0 (7)	C2—C1—C6—C7	1.2 (7)
C1—N1—C5—C4	0.8 (8)	C2—C3—C4—C5	−0.3 (8)
C1—C2—C3—C4	−0.3 (8)	C5—N1—C1—C6	178.7 (4)
C9—C14—C13—C12	0.3 (7)	C5—N1—C1—C2	−1.4 (6)
C9—C10—C11—C12	0.2 (8)		

### Dichlorido{(E)-4-methyl-N'-[1-(pyridin-2-yl)ethylidene]benzohydrazide}zinc(II) Hydrogen-bond geometry (Å, º)

**Table:**

*D*—H···*A*	*D*—H	H···*A*	*D*···*A*	*D*—H···*A*
N3—H3···Cl2^i^	0.87 (2)	2.40 (3)	3.214 (4)	156 (5)
C2—H2···Cl1^ii^	0.93	2.93	3.649 (5)	136
C4—H4···Cl1^iii^	0.93	2.98	3.888 (6)	165

Symmetry codes: (i) −*x*+1, −*y*+1, −*z*+1; (ii) *x*, *y*−1, *z*; (iii) −*x*+1, −*y*+1, −*z*.
